# What Makes an AI Persuasive? The Interaction Effect of AI Shopping Assistants and Algorithmic Recommendation Cues on Consumers’ Organic Food Purchase Intention

**DOI:** 10.3390/foods15172965

**Published:** 2026-08-24

**Authors:** Sinan Li, Kai Chen

**Affiliations:** School of Economics and Management, Beijing Forestry University, Beijing 100083, China; lisinan@bjfu.edu.cn

**Keywords:** organic food, purchase intention, analytic AI, empathic AI, algorithmic recommendation cues, perceived information validity, green trust

## Abstract

Promoting consumers’ organic food purchases is an important way to drive the green transformation of the demand side of food consumption and achieve sustainable development. This study, through two studies, examines the interactive effects and psychological mechanisms of different types of AI shopping assistants (analytic AI vs. empathic AI) and algorithmic recommendation cues (item-referent cues vs. user-referent cues) on consumers’ organic food purchase intention. The findings indicate that the two have an interaction effect, with analytic AI combined with item-referent cues being more effective, while empathic AI matched with user-referent cues is more effective. Further analysis revealed that perceived information validity and green trust each play a mediating role, forming a chain mediation path. Specifically, perceived information validity positively influences green trust, and green trust further positively influences purchase intention. This work reveals the matching mechanism between AI source characteristics and algorithmic recommendation cues, expands the application of Cue Utilization Theory and Signaling Theory in AI-driven organic food consumption contexts, and provides practical insights for companies to develop differentiated AI assistant configurations, recommendation information design, and green trust cultivation strategies.

## 1. Introduction

Climate change, environmental pollution, and ecosystem degradation have made the sustainable transformation of food systems a global issue. Food production, processing, and consumption are both affected by climate change and are significant sources of greenhouse gas emissions [1]. Research estimates that global food systems produce approximately 18 Gt CO_2_ equivalent annually, representing 34% of all emissions of greenhouse gases caused by human activities [2]. By forming demand-side pull through consumption choices and promoting organic production that reduces the use of synthetic pesticides and fertilizers, it can provide an important pathway for reducing agricultural nonpoint source pollution, protecting biodiversity, and advancing food system emissions reduction [3]. Simultaneously, improvements in living standards and increased awareness of health, food safety, and quality have led consumers to focus not only on the basic functions of food but also on its production methods and environmental impact [4,5]. In the U.S. market, organic food sales exceeded $60 billion in 2022, accounting for approximately 6% of total food sales, and over 80% of households had purchased organic food [6]. The demand for environmental governance and consumption upgrades thus form a dual driving force, making how to effectively promote consumers’ purchase of organic food an important issue in sustainable marketing and consumer behavior research.

However, organic food purchases have significant positive externalities. Consumers need to individually bear the price premium, while the benefits of reduced chemical inputs, improved ecological environment, and enhanced public health are shared by society as a whole [7,8]. More importantly, organic food has typical credence and good attributes, and its production process, environmental impact, and certification compliance are usually not directly verifiable by consumers before purchase or even after consumption [9]. Consumers therefore need to judge the credibility of certification labels and producers’ commitments based on limited information [10,11]. Higher prices, lack of knowledge about certifications, and ambiguous organic claims can further exacerbate information asymmetry and perceived uncertainty [12]. Greenwashing may even trigger green skepticism, causing consumers to worry that companies are exploiting their informational advantage for opportunistic behavior [13]. These elements negatively affect customers’ intentions to make purchases [14]. Compared to price subsidies or channel restructuring, information intervention has the advantages of being simple to implement, having low marginal costs, and being scalable. Large-sample choice experiments have shown that providing information labels related to organic food can increase consumers’ willingness to pay by approximately 40%, with a 50% increase among high-trust consumers, indicating that the synergistic construction of information and trust is particularly important for the promotion of organic food [15]. Therefore, alleviating the uncertainty of organic attributes and increase purchase intention through low-cost, scalable information intervention techniques constitutes a crucial component of encouraging sustainable food consumption.

Existing research has examined the role of information interventions from the perspectives of advertising appeals, information presentation, and message framing, finding that advertising information can influence sustainable decision-making through cognitive processing, environmental values, and normative beliefs. However, its effectiveness depends on the content of the information and the characteristics of the audience [16]. Research on organic food has found that self-interest and altruistic appeals need to match different product attributes to enhance processing fluency and purchase intention [17]; additionally, specific combinations of environmental appeals and gain–loss frames can also produce differentiated persuasive effects [3]. Nevertheless, the primary emphasis of these investigations is textual frameworks such as “Gain–Loss,” “Positive–Negative,” or “Egoism–Altruism,” and rarely examines the reasons behind the organization of recommendation evidence, especially in practical contexts where AI and algorithms are widely applied in marketing and consumer behavior interventions. In consumer scenarios where algorithms provide recommendation information, the rationale for recommendations can either point to product attributes and comparisons between goods or to the preferences of the consumer themselves or similar users, forming item-oriented and user-based algorithmic recommendation information, respectively [18]. The recommendation effects of these two different types of information have been validated in the context of purchasing general products [19], but the differential impact of these information cues in the context of purchasing organic food still lacks systematic testing. At the same time, AI shopping assistants have deeply integrated into search, consultation, and shopping decision-making scenarios, evolving into different styles and types of AI assistant types. For example, AI with emotional understanding capabilities can enhance consumers’ trust in their recommendations and their willingness to delegate decision-making [20]. However, current AI marketing research mostly compares the effectiveness of AI and humans as the main comparison logic [21,22]. Some studies also point out that the roles of robots, intelligent chatbots, and algorithms are not the same [23]. Research on AI in food consumption contexts has examined both sensory evaluation and consumer responses. Technologically, AI methods combining instrumental and human sensory data (e.g., electronic noses/tongues, computer vision, machine learning) can assess food attributes and predict consumer responses [24,25]. From a consumer perspective, Nozawa et al. [26] found that AI-provided food and restaurant services are evaluated less favorably than human-provided ones, especially in luxury restaurants. Zhang and Zhou [27] further show that algorithmic recommendations are more accepted for distal senses (vision, hearing) than proximal ones (taste, smell, touch), as algorithms are perceived as better suited to the former. Overall, AI influences both food evaluation and consumer experience, but prior research mainly contrasts AI’s and humans’ effects [28], lacking sufficient explanation of how AI capability orientations should be matched with different recommendation evidence in organic food decisions. However, as different types of AI are widely applied in interventions for consumer purchase decisions, what kind of algorithmic recommendation cues should be conveyed by AI and whether they can achieve a match to enhance organic food purchase intention remain to be answered.

To fill the aforementioned gap, this work examines the interactive effects of AI shopping assistants and algorithmic recommendation cues on willingness to buy organic food. Specifically, according to the research by Huang and Rust [29], we distinguish analytic AI from empathic AI according to the capability orientation made salient in the shopping interaction. Analytic AI foregrounds data processing, comparative analysis, logical reasoning, and objective judgment, thereby signaling competence. Empathic AI foregrounds the understanding of consumer needs and concerns, emotionally responsive communication, and perspective-taking, thereby signaling warmth. This distinction concerns the focal capability and communication orientation presented to consumers rather than mutually exclusive underlying technical functions. In addition, drawing on the research by Gai and Klesse [18], we categorize algorithmic recommendation cues into item-referent and user-referent cues, respectively emphasizing the similarity to similar products and the similarity to other users’ preferences. We hypothesize that analytic AI matching with item-referent cues is more effective in enhancing purchase intention, while empathic AI combined with user-referent cues yields even better results. Furthermore, we reveal the underlying mechanisms of this interaction from the perspectives of cognitive evaluation and green relationships, suggesting that perceived information validity and green trust play mediating roles. Perceived information validity reflects consumers’ judgment of the rationality and accuracy of the information, while green trust reflects consumers’ trust in the organic attributes and green commitments of the product. Based on Signaling Theory, we hypothesize a theoretically ordered indirect association in which perceived information validity positively influences green trust, which subsequently influences purchase intention. This proposed ordering is evaluated as a serial mediation model.

This work has theoretical contributions. First, it advances the research on AI shopping assistants from a static comparison of which type of AI is more effective to a dynamic matching perspective of which AI is more effective under which algorithmic recommendation cues, indicating that different AI shopping assistants do not have an absolute advantage independent of the recommended content. Second, this work expands the application of Cue Utilization Theory in the field of sustainable food consumption behavior, linking AI capabilities and warmth source characteristics with algorithmic recommendation cues. It reveals how consumers integrate information source cues with recommendation content cues, thereby establishing a theoretical connection between the Stereotype Content Model and Cue Utilization Theory. Third, this work reveals the transformation process of algorithmic signals based on Signaling Theory. The recommended information cues need to be judged as accurate, reasonable, and effective before they can be transformed into trust in organic attributes and green commitments, which in turn affects purchase intention. In practice, this work can provide organic food marketers with AI selection and information design guidelines, while also enhancing the validity of recommended information through source disclosure and traceable information, continuously building green trust and increasing consumers’ organic food purchase intention. As a result, the central objective of this research is to determine when and why different AI shopping assistants enhance consumers’ organic food purchase intentions. Across two randomized experiments, we test whether AI type interacts with item- versus user-referent recommendation cues and whether perceived information validity and green trust explain this effect sequentially.

## 2. Literature Review and Theoretical Framework

### 2.1. Analytic AI and Empathic AI

With the advancement of generative AI, machine learning, and natural language processing, AI assistants have gradually evolved from technical tools performing simple queries and automated customer service tasks to interactive marketing entities capable of participating in consumer information searches, product comparisons, solution evaluations, and purchase decisions. In online and mobile retail environments, AI shopping assistants can simulate natural language interactions between sales personnel and consumers, and by providing personalized information and product recommendations, they reduce the difficulty consumers face in understanding products and making choices [30]. Similarly, AI recommendation systems can influence consumer behavior decisions through trust in recommenders and products, indicating that AI shopping assistants have deeply integrated into the consumer’s product discovery and selection process [31]. This type of purchase activity, which uses digital assistants as the interaction interface, is also summarized as conversational commerce, where consumers obtain information, suggestions, and advance their purchase decisions through interactions with AI assistants [32]. As its application scope expands, researchers have begun to classify AI shopping assistants from different dimensions. From the perspective of presentation form, they can be categorized into physical robots, text or voice chatbots, and backend algorithms [23]. From the perspective of autonomy and relational roles, they can be categorized into AI assistants that primarily respond to user commands, AI agents that can autonomously plan and execute tasks, and AI companions that are centered around maintaining ongoing emotional relationships [33]. From the perspective of the intelligent capabilities relied upon to complete tasks, AI may be further divided into machine, feeling, and thinking AI, focusing on data processing, logical reasoning, and decision optimization, as well as understanding and responding to consumers’ emotional and social needs [34]. Given that AI shopping assistants need to help consumers make product judgments and also understand consumer needs through interpersonal communication, this paper further focuses on the two dominant orientations they present in interactions, namely analytic AI and empathic AI. This classification is also consistent with consumers’ social perception of AI assistants, who typically evaluate AI assistants’ responses along two fundamental dimensions: competence and warmth. The former reflects whether the AI is accurate and capable, while the latter reflects whether the AI is sincere and able to establish emotional connections [35].

In this research, we define AI shopping assistants as AI systems embedded in online or mobile shopping environments that interact with consumers through natural language and utilize product data, user information, and algorithmic analysis to provide product search, comparison, explanation, and personalized recommendations, thereby assisting consumers in making purchasing decisions. On this basis, analytic AI assistants refer to shopping assistants that primarily emphasize analytical abilities, logical reasoning, and objective judgment in shopping interactions. Therefore, analytic AI primarily conveys to consumers the competence characteristic of the assistant’s ability to make accurate judgments [36]. In contrast, empathic AI assistants are shopping assistants that primarily emphasize the ability to understand needs and respond emotionally. It not only identifies the preferences explicitly expressed by consumers but also pays attention to their potential concerns, value appeals, and emotional states. By acknowledging consumers’ feelings, using caring language, and explaining recommendations from the consumers’ perspective, it creates an experience where consumers feel understood and valued [37]. This type of AI typically includes functions such as emotion recognition, empathetic care, and appropriate emotional responses. These capabilities help bridge the social and emotional experience gap between AI and human service personnel [38]. It should be noted that analytic AI is not completely devoid of warmth, and empathic AI is not without data analysis capabilities; both can use the same underlying models and recommendation algorithms. The differences mainly manifest in the roles, communication styles, and ability signals that are prominently displayed during interactions [39], and both can promote purchase intention.

### 2.2. Item-Referent Cues and User-Referent Cues

As smart shopping platforms continuously expand their range of product selections, algorithmic recommendation has become an important tool for companies to reduce consumer search costs and influence purchasing decisions. Algorithmic recommendation systems typically process data such as consumers’ browsing, clicking, and purchasing records, as well as product attributes, to predict consumers’ preferences for different products and subsequently filter and rank the recommendation results [40,41]. Algorithmic recommendation not only helps consumers discover products with higher value, lower prices, or better alignment with their preferences, but also influences their search methods and purchase probabilities [42]. Moreover, the informational cues of recommendations further influence consumers’ search intensity, search efficiency, and purchasing behavior, indicating that the influence of algorithmic recommendations on the choices made by customers depends not only on the recommendation results generated in the backend but also on how the platform frames and cues these results to consumers [43]. From a technical implementation perspective, common collaborative filtering methods in algorithmic recommendation systems include user collaborative filtering, which generates recommendations based on similar consumer preferences, and item collaborative filtering, which generates recommendations based on the associations or similarities between products [19]. However, in reality, algorithmic recommendation systems often utilize both consumer and product data. Even if the data sources and algorithms are exactly the same, platforms can still present it as “this product is similar to the ones you have browsed” or “other similar consumers have also purchased this product” [18]. Therefore, starting from the recommendation reasons that consumers can directly perceive, this paper further focuses algorithmic recommendations into two types: item-referent and user-referent cues.

Combining the recommendation framework with explainable recommendation research, drawing on the findings of Gai and Klesse [18], this work defines algorithmic recommendation cues as brief explanatory information provided by AI shopping assistants when presenting recommendation results, explaining why the results are worth considering and indicating the evidence base on which the recommendations are primarily built. These cues provide consumers with perceptible judgment bases to understand the previously invisible algorithmic inference process [44]. Specifically, item-referent cues direct consumers’ attention to the product itself and the relationships between different products, emphasizing evidence at the product level such as category consistency, attribute similarity, or functional matching. This type of cue primarily conveys “product matching,” highlighting the attribute basis and comparative logic of the recommendation [45]. In contrast, user-referent cues direct consumers’ attention to the preference connections between consumers and users, emphasizing the browsing, purchasing, or usage experiences of similar consumers. These cues not only imply that the recommended products match the target consumer but also convey a “preference matching” or “taste matching” between the consumer and the referent user, thereby exhibiting more prominent social reference and individual relevance characteristics [18]. Related research has found that when consumers’ shopping goals are relatively vague, user-referent framing is more likely to be effective; whereas when the goals are more defined, item-referent framing is more effective [19]. It can be seen that the core difference between the two types of cues does not necessarily lie in the algorithms being different, but in the different directions of the evidence highlighted by the recommendation explanations.

### 2.3. The Interaction Effect of AI Shopping Assistants and Algorithmic Recommendation Cues

AI shopping assistants and algorithmic recommendation cues do not independently influence consumer decisions; rather, they jointly form a combination that shapes consumers’ understanding and evaluation of recommendation information. According to the classification of AI capabilities, analytic AI excels in thinking, emphasizing data processing, pattern recognition, and logical reasoning; empathic AI highlights feeling, with the ability to recognize and understand consumer emotions [29]. From the Stereotype Content Model (SCM) perspective, consumers typically evaluate social actors along the two basic dimensions of competence and warmth, and this evaluation method is equally applicable to AI. Analytic AI is more likely to highlight characteristics of professionalism, precision, and competence, while empathic AI is more likely to emphasize understanding, care, and warmth [46,47]. Simultaneously, item-referent cues present the basis of recommendations as the similarity of attributes or functions between products, while user-referent cues emphasize the similarity of preferences or tastes between other users and intended customers [18]. Therefore, AI types determine how consumers understand what assistants are good at, while algorithmic recommendation cues explain the basis of that information; considering both together can fully explain the persuasive effect of recommendations. We believe that the information source kinds and the information’s substance will interactively influence organic food purchase intention [48].

Cue Utilization Theory provides the core theoretical explanation for the aforementioned interaction effect. The theory posits that when consumers cannot directly observe or verify the true quality of a product, they will use a series of available product and situational cues to form quality judgments [49]. Importantly, consumers do not process each cue in isolation but rather integrate multiple cues; different cues may reinforce, substitute, or counteract each other, so their combined effect is not necessarily equal to the simple sum of their individual effects [50]. In this study, AI shopping assistants serve as an information source cue, reflecting the assistants’ capabilities and relational orientation; algorithmic recommendation cues, on the other hand, serve as a recommendation basis cue, indicating whether the recommendations are based on product similarity or user similarity. An item-referent cue is an information cue that points to product attributes; a user-referent cue, on the other hand, is an information cue that points to social comparison and similar preferences. When the source cue and the recommendation basis cue point to the same meaning dimension, they can match and validate each other. One cue answers why the AI is capable of making such a recommendation, while another cue answers why the basis for the recommendation is trustworthy, thereby enhancing the overall consistency of the cue combination [28]. Due to the trust attributes of organic food, such as pesticide residues, production methods, and environmental performance, which consumers find difficult to fully verify even after purchase, the consistency of cues is particularly important for their judgment [51]. Thus, the matching of the two cues can effectively enhance purchase intention.

Specifically, analytic AI has a high cognitive match with item-referent cues. Analytic AI emphasizes data analysis, computational accuracy, and objective judgment capabilities, thereby primarily conveying competence cues [22]. Item-referent cues direct consumers’ attention to comparable attributes between products, and when combined, the recommendation content aligns with the core competencies of the recommender. Item-referent cues provide judgment criteria at the product level, while the capability cues of analytic AI indicate that these criteria are obtained through the comparison and analysis of historical browsing data. Consumers are therefore more likely to believe that recommendations are based on information directly related to product quality, thereby increasing the diagnosticity of the cues and subsequently enhancing purchase intention [19]. Although organicness itself is a trust attribute that is difficult to directly verify, item-referent cues can transform these unobservable attributes into relatively verifiable information through certification and traceability data, and analytic AI happens to provide a competence endorsement for this analytical process [52]. Therefore, under the conditions of analytic AI, item-referent cues are more effective than user-referent cues in enhancing organic food purchase intention.

In contrast, empathic AI has a higher relational match with user-referent cues. Empathic AI conveys goodwill and relationship-oriented cues by understanding consumer emotions, addressing individual needs, and providing supportive responses; user-referent cues, on the other hand, emphasize that similar consumers like or choose the product, with their persuasiveness stemming from consumer similarity and social identification [18]. Wien and Peluso [22] pointed out that recommenders with human-like characteristics are more likely to activate consumers’ mentalizing and self-referencing, and promoting purchases. Choi and Kim [19] also found that when it is difficult to collect objective information to evaluate a product before purchase, consumers are more likely to rely on user-based recommendations. The understanding and care conveyed by empathic AI precisely validate the core premise implied by user-referent cues, namely that these shopping assistants can understand consumers like me and translate others’ experiences into recommendations relevant to me. This is particularly important for organic food choices that simultaneously involve health pursuits, environmental values, and moral identity expression. The consistency between this information source cue and social recommendation basis can enhance consumers’ sense of being understood and self-referential judgment, making user-referent cues perceived as more relevant and effective information, thereby increasing consumer acceptance [53]. In contrast, when empathic AI provides item-referent cues, its warmth and empathetic characteristics represent the advantages of relational source cues, which results in a certain mismatch with the product characteristic recommendation basis. Therefore, under the conditions of empathic AI, user-referent cues are more effective than item-referent cues in enhancing organic food purchase intention. Through the above analysis, the research hypothesis can be put forth:

**H1.**  *An interaction effect exists between AI shopping assistants and algorithmic recommendation cues, specifically*.

**H1a.**  *When analytic AI conveys item-referent cues (vs. user-referent cues), consumers show higher organic food purchase intention*.

**H1b.**  *When empathic AI conveys user-referent cues (vs. item-referent cues), consumers show higher organic food purchase intention*.

### 2.4. The Mediating Role of Perceived Information Validity

Perceived information validity refers to the subjective evaluation formed by consumers regarding whether specific information can serve as a valid basis for judgment and decision-making, primarily manifested in consumers’ belief that the information possesses accuracy, authenticity, and trustworthiness [54]. It reflects not whether the information is completely correct in an objective sense, but whether consumers believe that the information is consistent with their existing knowledge, situational evidence, and the source of the information, thereby supporting their decision-making. Relevant research indicates that information recipients will simultaneously assess the reasonableness and credibility of the information and sources, and based on these two factors, form a judgment of information validity [55], which in turn affects the persuasive effect of the information [56]. This mechanism is particularly important in the context of organic food consumption. Due to the health, safety, and environmental friendliness attributes of organic food being typical trust goods, consumers find it difficult to directly verify related green and organic claims even after purchase and use. Their decisions heavily rely on alternative evidence such as certifications, product descriptions, and recommendations [10]. When consumers view an information from AI shopping assistants as accurate, reasonable, and trustworthy, this information can reduce the uncertainty and perceived risk associated with organic attributes, thereby enhancing consumers’ confidence in their value judgment of the product and increasing the likelihood of accepting recommendations and making purchases [57,58]. Conversely, if the information is perceived to lack authenticity or a reliable basis, consumers may become skeptical of the recommendations. This is especially true when erroneous information comes from generative AI, which can lower consumers’ evaluations of the credibility and accuracy of an information, thereby weakening their behavioral intentions [59]. In high-uncertainty decision-making, consumers’ acceptance of AI recommendations also relies more on perceived accuracy judgments [60]. Therefore, perceived information validity constitutes the key internal factor in the transformation of external recommendation cues into purchase intention.

Specifically, algorithmic recommendation cues inform consumers about the reference basis on which the information is based. Item-referent cues emphasize the similarity of the recommended product to other products in terms of attributes, functions, or categories, while user-referent cues highlight the connection between the preferences and choices of users similar to the consumer [61]. Consumers do not evaluate recommendation information in isolation; instead, they integrate the capability features presented by AI shopping assistants with the types of evidence indicated by algorithmic recommendation cues. Consistency between the source and the content of the information can enhance the information’s rationality and perceived validity, while inconsistency may lead to consumer skepticism toward the recommendation information [55,62]. Under item-referent cue conditions, the rationale for recommendations is primarily based on product attribute comparisons and category matching. The analytical capabilities, objectivity, and algorithmic precision demonstrated by analytic AI align with this product-level evidence structure, making it easier for consumers to infer that the recommendation results stem from systematic product comparisons, thereby considering the recommendation information to be more accurate and effective [63,64]. Under user-referent cue conditions, the effectiveness of recommendations depends on whether AI can accurately understand individual consumer needs and reasonably map the preferences of similar users to the target consumer. The understanding and care embodied by empathic AI align with the user-level inference basis, thereby enhancing the individual relevance and perceived validity of the recommendation information [65,66], which in turn increases consumers’ acceptance of AI recommendations [67]. Therefore, under the two types of recommendation cues, AI shopping assistants alter consumers’ perception and judgment of information validity, subsequently affecting their organic food purchase intention. The following hypothesis is put out in light of the analysis presented above:

**H2.**  *Perceived information validity mediates the effect of AI shopping assistants and algorithmic recommendation cues on organic food purchase intention*.

### 2.5. The Mediating Role of Green Trust

Customers’ willingness to rely on a product, service, or brand based on their perception or expectation of its environmental performance, credibility, capacity, and goodwill is known as green trust [68]. In this study, green trust specifically refers to consumers’ trust that the recommended organic food can genuinely fulfill environmental commitments, maintain organic attributes, and achieve the expected environmental benefits. The production methods of organic food, the use of pesticides and fertilizers, certification standards, and actual environmental benefits possess typical trust attributes. Even after purchasing and consuming, consumers find it difficult to independently verify these aspects, making them susceptible to information asymmetry, false green claims, and “greenwashing” risks [69]. In this context, green trust can serve as a psychological mechanism to reduce uncertainty and relational risk [70]. When consumers believe that the organic attributes and environmental commitments of a product are genuine and reliable, they will reduce their concerns about false advertising and product failure, and be more confident that purchasing the product can simultaneously achieve health and environmental goals. They are therefore more inclined to follow AI suggestions and develop an intention to buy organic food [71]. Therefore, we believe that when external information influences purchasing intention, green trust acts as a mediator [72].

From the perspective of information processing, the capability characteristics of AI shopping assistants will influence whether consumers are willing to trust the evidence provided by algorithmic recommendation cues, and further transfer their trust in the recommendation information to the purchase intention of the recommended organic food [28]. Under item-referent cues, the recommendation information focuses on comparing the environmental attributes of products; the analytical capabilities and objectivity demonstrated by analytic AI align with this product-level information, making it easier for consumers to believe that AI can systematically identify and compare the organic attributes of products. This clear, specific, and fact-based information is more likely to form green trust based on competence and reliability [73]. In contrast, under user-referent cues, the recommendations are primarily based on and the choices of similar consumers. At this time, the warmth and empathy capabilities embodied by empathic AI can enhance the sense of relational connection between consumers and similar users, reduce their suspicion of manipulative or opportunistic motives behind the recommendations, and form a green trust based on goodwill and value consistency [74], thereby increasing the willingness to buy organic food [75]. Consequently, the following hypothesis is put forward:

**H3.**  *Green trust mediates the effect of AI shopping assistants and algorithmic recommendation cues on organic food purchase intention*.

### 2.6. The Serial Mediating Effect of Perceived Information Validity and Green Trust

Perceived information validity and green trust, respectively, represent the cognitive evaluation and trust formation process of consumers when processing AI recommendation information. Perceived information validity mainly reflects consumers’ evaluation of whether the recommended information is accurate, true, and reasonable; green trust, on the other hand, is the trust expectation formed by consumers based on this information regarding whether organic food and its suppliers can reliably fulfill their commitments to environmental friendliness, food safety, and health value. Due to the typical trust attributes of organic food, consumers need to rely on external signals such as certifications, product ingredients, environmental claims, and others’ experiences to assess its true value [51,76].

According to Signaling Theory, in situations of information asymmetry, consumers use observable signals to infer the quality of products that cannot be directly observed. Theoretically, the behavioral influence of these signals depends on whether consumers regard them as clear, credible, and useful [77]. Accordingly, product data, user preferences, and the experiences of similar consumers conveyed by AI shopping assistants may contribute to perceived information validity. A more favorable validity judgment may lead to stronger confidence that the product’s organic attributes and environmental commitments are genuine and reliable, further promoting purchase intention [78,79]. In the context of organic food consumption, information validity can reduce consumers’ uncertainty about health, food safety, and environmental benefits, and convert these perceived benefits into purchase intention [6]. Green trust subsequently further reduces consumers’ concerns about false environmental claims, greenwashing, and product performance uncertainty, making them more willing to bear the price premium and decision risk involved in purchasing organic food [80]. Together, they sequentially form a continuous process. On this theoretical basis, we propose an ordered indirect association from perceived information validity to green trust and to purchase intention. Importantly, this ordering is derived from the theoretical distinction between evaluating recommendation information and relying on the product’s green commitments. The empirical analysis tests whether the observed data are consistent with this theoretically specified ordering.

In this work, this chain reaction can occur under two types of algorithmic recommendation cues. Under item-referent cue conditions, product ingredients, certification standards, and comparisons with similar products constitute product-level signals for consumers to judge the value of organic food [10]. The analytical capabilities and computational accuracy demonstrated by AI are consistent with the evidence sources for comparing product attributes. As long as this evidence is based on sufficient factual grounds and product information disclosure, consumers are more likely to consider the recommendation information as valid, thereby believing in the product’s organic attributes and green commitments and forming a purchase intention [81]. Under user-referent cues, the choices of similar consumers, individual preference matching, and usage experience constitute social signals. Empathic AI, by demonstrating understanding and empathy, makes it easier for consumers to believe that the system accurately understands their needs and that the recommended conclusions align with their personal interests, thereby enhancing the perception of information effectiveness and subsequently forming green trust, which in turn increases purchase intention [82]. Based on the above analysis, the following hypothesis is proposed:

**H4.**  *Perceived information validity and green trust serve as a serial mediator in the effect of AI shopping assistants and algorithmic recommendation cues on organic food purchase intention*.

**Figure 1:**
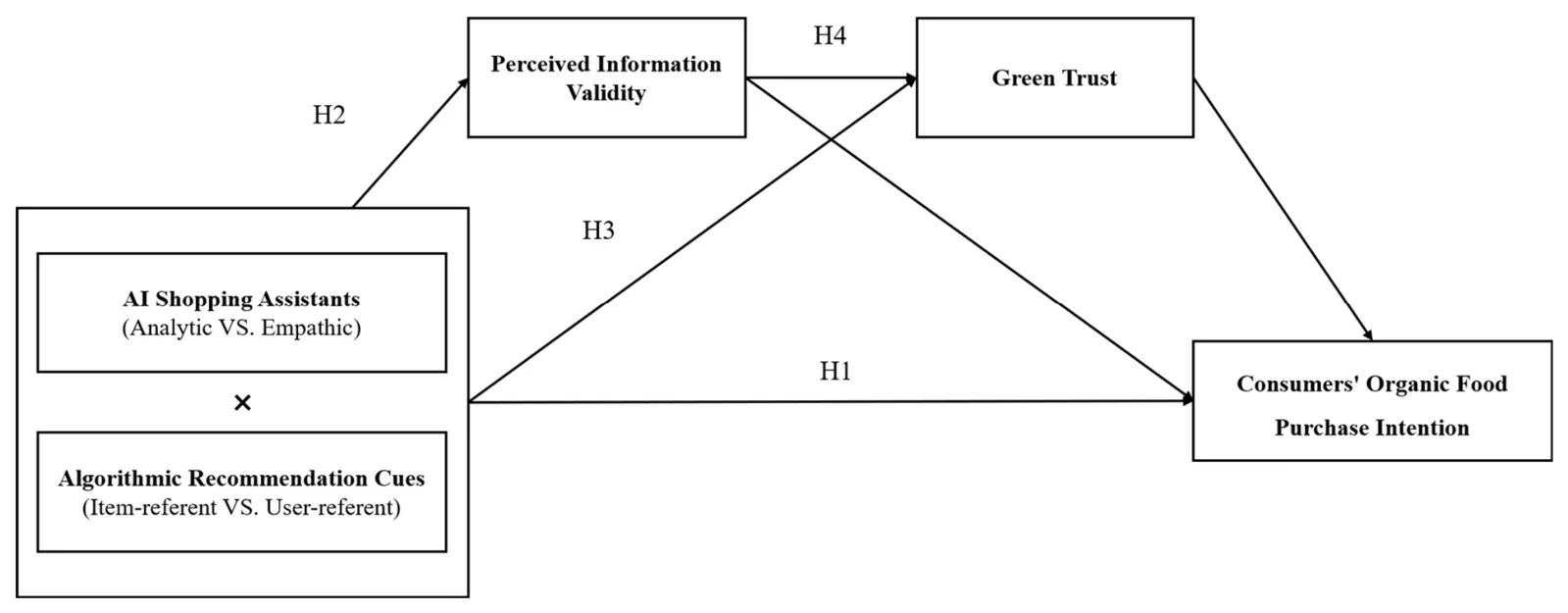
Conceptual model.

In order to present the relationships between the variables and the research hypotheses, we have drawn a conceptual model (Figure 1).

## 3. Study 1

### 3.1. Pilot Test

Determining the stimuli to be utilized in the formal experiment is the goal of the pilot test. First, we reviewed relevant reports and statistical data published by the FAO and found that the reports categorize foods that provide daily energy needs for humans into nine categories: cereals, fats and oils, sugar, fruit and vegetables, roots, tubers and pulses, meat, dairy and eggs (excl. butter), beverages and other, and fish and seafood [83,84]. Furthermore, we consulted the “Guidelines for the Production, Processing, Labeling, and Marketing of Organically Produced Foods (CXG 32-1999)” [85] established and published by the FAO/WHO Joint Food Standards Program’s Codex Alimentarius Commission (CAC). We found that these guidelines do not have specific standards for aquaculture products and wild-caught seafood, as they consider these products not to fall within the clearly established scope of organic production [85]. Therefore, fish and seafood are not included in this research.

Next, we identified eight specific foods commonly found in daily life as alternatives corresponding to the eight categories of food mentioned above through focus group discussions. These include organic rice, organic peanut oil, organic white sugar, organic apples, organic potatoes, organic pork, organic pure milk, and organic tea leaves. Subsequently, we conducted a questionnaire survey to measure consumers’ familiarity with these products, their daily consumption frequency, and their propensity to buy. All items used 7-point Likert scales, which helps reduce the barriers consumers face in judging and understanding product attributes in formal experiments. Seven categories provide fine-grained discrimination without imposing the cognitive burden associated with very long scales. Prior research indicates that reliability, validity, and discriminating power generally improve up to approximately seven response categories and that five- and seven-point formats yield broadly comparable distributional properties. We also retained the same response format across the pilot test and formal experiments to maintain measurement consistency and followed the response formats of the original scales where applicable. We recruited 110 participants for the pilot test (N_female_ = 68.18%, M_age_ = 32.06 SD = 10.70, see Table 1 for details). The participants’ age intervals reported in Table 1 were used solely to describe the demographic composition of the pilot test sample; they were not sampling quotas, experimental conditions, or analytical groups. Accordingly, no comparisons or statistical inferences were made across the age categories. We listed these eight foods and asked participants to report their familiarity with these products (1 = very unfamiliar, 7 = very familiar, adapted from Huang et al. [28]), their frequency of daily consumption (1 = never, 7 = always, adapted from Zhao et al. [86]), and their likelihood of purchase (1 = very unwilling, 7 = very willing, adapted from Li et al. [87]).

Regarding selection criteria, participants were eligible for the pilot test if they were at least 18 years old and regularly participated in household food purchasing. These criteria ensured that respondents had direct experience with daily food choices. A total of 110 eligible participants completed the pilot. Because the pilot was designed primarily for descriptive stimulus selection, product selection was based on convergent evidence from familiarity, consumption frequency, and purchase likelihood. As a conservative sensitivity check, with α = 0.05 (two-tailed), N = 110 provides 80% statistical power to detect a minimum detectable effect of 0.27 in a one-sample or paired mean comparison. Thus, the pilot was sufficiently sensitive to “small-to-moderate” participant differences relevant to stimulus screening. In addition, the half-widths of the 95% confidence intervals around the 24 product-level means ranged from 0.16 to 0.32 points on the 7-point scales (median = 0.25), indicating adequate estimation precision for the intended stimulus-screening purpose.

The results showed that the participants’ familiarity with the above eight products, ranked from high to low, is as follows: M_organic pure milk_ = 6.08, M_organic potatoes_ = 5.78, M_organic rice_ = 5.76, M_organic apples_ = 5.68, M_organic peanut oil_ = 5.58, M_organic pork_ = 4.86, M_organic white sugar_ = 4.05, M_organic tea leaves_ = 4.02. Daily consumption frequency ranked from high to low: M_organic pure milk_ = 5.52, M_organic rice_ = 5.19, M_organic potatoes_ = 5.06, M_organic peanut oil_ = 4.91, M_organic apples_ = 4.90, M_organic pork_ = 4.12, M_organic white sugar_ = 3.21, M_organic tea leaves_ = 3.20. Purchase likelihood from high to low: M_organic pure milk_ = 6.15, M_organic potatoes_ = 6.03, M_organic rice_ = 5.96, M_organic apples_ = 5.81, M_organic peanut oil_ = 5.77, M_organic pork_ = 5.37, M_organic white sugar_ = 4.46, M_organic tea leaves_ = 4.27. Product selection followed two complementary criteria. First, the formal stimuli were required to score consistently high across familiarity, daily consumption frequency, and purchase likelihood. Organic pure milk ranked first on all three dimensions (Mfamiliarity = 6.08, Mfrequency = 5.52, Mpurchase likelihood = 6.15). Organic potatoes ranked second in familiarity (M = 5.78) and purchase likelihood (M = 6.03) and third in consumption frequency (M = 5.06). Both products therefore provided familiar and behaviorally relevant contexts for the formal experiments. Considering the reported results of familiarity, consumption frequency, and purchase likelihood, we selected organic pure milk and organic potatoes as the stimuli for the formal experiments in Study 1 and Study 2, respectively. Study 2 was designed as a conceptual replication using a different food category and product form. According to the FAO’s report, organic pure milk represents the “dairy and eggs” category, whereas organic potatoes represent the “roots, tubers and pulses” category. By retaining the factorial design, manipulations, and outcome measure while varying the focal product, we sought to examine whether the predicted interaction pattern could be reproduced beyond a single organic food stimulus. Based on this, we designed the experimental materials, which can reduce cognitive barriers for consumers during the experiment [88]. For detailed information, see Table 2.

### 3.2. Participants and Design

The purpose of Study 1 is to evaluate the interaction impact of AI shopping assistants and algorithmic recommendation cues, specifically to test Hypothesis 1. We adopt a 2 (AI shopping assistants: analytic AI vs. empathic AI) × 2 (algorithmic recommendation cues: item-referent cues vs. user-referent cues) between-subject experimental design. We conducted a priori power analysis to determine the required minimum sample size. Leveraging G*Power 3.1 to calculate the sample size, with an F-test, an effect size of 0.25, α error level is 0.05, and a statistical power of 0.8, the total sample size was 128 [89].

In Study 1, we used organic pure milk as the formal experimental stimulus, manipulating different AI shopping assistants and algorithmic recommendation cues to examine whether participants’ organic food purchase intentions showed significant differences after being exposed to different material stimuli. In the manipulation of AI shopping assistants, we used the same image for different experimental conditions and distinguished the characteristics of different assistants through textual descriptions. For example, the analytic AI stated, “I excel at organizing data, conducting comparative analysis, and making logical judgment.” While the empathic AI stated, “I excel at understanding consumers’ needs and feelings when purchasing products” (see the Appendix A), this helps eliminate the noise caused by the experimental manipulation of preferences and aversions toward different personas. For the design of algorithmic recommendation cues, two categories were used: “item-referent” and “user-referent.” Item-referent cues emphasize foods similar to products the consumer has previously shown interest in or browsed, such as “According to the algorithm recommendation system, this organic pure milk has been found to be similar to other organic foods you have recently shown interest in.” User-referent cues emphasize similar consumer purchase choices and preferences, such as “According to the algorithm recommendation system, other consumers with similar consumption preferences to yours have been identified.” (see the Appendix A).

To evaluate the experimental manipulation’s efficacy, we designed manipulation check questions targeting the variables. After receiving the experimental material stimuli and completing the questions related to purchase intentions, participants need to further answer these manipulation check questions. All items are designed using a 7-point scale (1 = strongly disapprove, 7 = strongly approve). The questions about AI shopping assistants include: (1) “The AI shopping assistant mentioned above excels at systematically evaluating product features;” (2) “The AI shopping assistant mentioned above specializes in data organization and information analysis;” (3) “The AI shopping assistant mentioned above excels at understanding emotions and feelings;” (4) “The AI shopping assistant mentioned above specializes in identifying consumer concerns and perceiving needs.” The topics of algorithmic recommendation cues include: (1) “The information above is recommended according to similar products I have recently viewed;” (2) “The information above highlights similarities to products I have previously browsed;” (3) “The information above is recommended according to the preferences and choices of consumers similar to me;” (4) “The information above highlights the preferences of consumers similar to me regarding this product.”

Participants for Study 1 were recruited through Credamo (a professional research platform). The study participation invitations were distributed to registered members, who voluntarily decided whether to participate. The sample for Study 1 should contain consumers who may encounter AI recommendations when purchasing food on shopping websites. Therefore, consumers of different genders, ages, education levels, and income groups were recruited without imposing population quotas that restrict sample breadth. Using organic pure milk, a food with high familiarity, makes the experimental context relevant to consumers. As shown in Table 3, the sample includes participants from various demographic categories.

Study 1, after obtaining informed consent, randomly assigned participants through the survey system with equal probability to one of the four experimental conditions. Multiple submissions and forwarding of the questionnaire are prohibited. A total of 280 questionnaires were collected. After excluding 9 patterned responses (such as all identical options and “Z”-shaped options) and 8 responses that met the criterion of too short completion time (60 s), 263 valid samples were retained (N_female_ = 58.17%, M_age_ = 29.72, SD = 9.13, N_analytic-item group_ = 67, N_analytic-user group_ = 65, N_empathic-item group_ = 66, N_empathic-user group_ = 65).

### 3.3. Procedure and Measures

At the beginning of the formal experiment, we set up a scenario and informed the participants to imagine themselves in that scenario. We presented the following text: “Please imagine that you intend to purchase pure milk and are browsing an online shopping platform. This platform will prioritize matching you with the nearest delivery station and deliver the food you ordered within half an hour. The platform has set up AI shopping assistants that can recommend products to consumers based on relevant information in the shopping system. At this point, the AI shopping assistants recommended an organic pure milk to you, and you saw the following information.” Then, participants were randomly assigned to one of four experimental conditions.

In this formal experiment, we supplemented information and introductions related to organic pure milk, including certification information, production method, ingredients and additives, traceability and environment, to enhance participants’ sense of authenticity and immersion. For example, we used the following statements: “Certified as an organic product, certification details are available for inquiry;” “The milk source comes from organic certified farms; the feed is grown organically, without the use of synthetic pesticides and fertilizers.” Participants, after receiving the material stimulus, need to respond to the 7-point scale regarding their purchase intention for the organic pure milk (1 = strongly disapprove, 7 = strongly approve): (1) “It is very likely that I will purchase the organic pure milk listed above;” (2) “I think the organic pure milk listed above is worth buying;” (3) “I would recommend the organic pure milk listed above to people I know;” (4) “Generally, I am willing to purchase the organic pure milk listed above.” (adapted from Li et al. [87]). Subsequently, participants answered manipulation check items, including judgments on AI shopping assistants and algorithmic recommendation cues. Finally, they answered questions related to demographic variables, including education level, age, gender, etc.

Regarding the methods of statistical testing, multi-item construct scores were computed as item means after internal consistency was evaluated using standardized Cronbach’s alpha. Manipulation checks used a paired-sample *t*-test to compare the two perceived AI dimensions within each AI condition and the two recommendation-cue dimensions within each cue condition. H1 was tested using a two-way ANOVA analysis with AI shopping assistant and algorithmic recommendation cue as between-subject factors. The interaction was followed by an independent-samples *t*-test comparing item- and user-referent cues separately within the analytic AI and empathic AI conditions. We reported partial eta squared for the interaction and Cohen’s d for the comparisons.

### 3.4. Results

We first examined whether the experimental manipulation was successful. The paired-sample *t*-test findings demonstrated that when consumers received information from an analytic AI, they were more likely to perceive AI shopping assistants as the analytic type rather than the empathic type (M_analytic_ = 5.93, SD = 0.61; M_empathic_ = 4.36, SD = 1.42, *p* < 0.001); conversely, when receiving information from empathic AI, participants were more likely to perceive AI shopping assistants as the empathic type (M_analytic_ = 4.80, SD = 1.48; M_empathic_ = 5.82, SD = 0.82, *p* < 0.001). Regarding judgments of algorithmic recommendation cues, when participants received information emphasizing item-referent cues, they rated the item dimension higher (M_item_ = 5.97, SD = 0.92; M_user_ = 4.52, SD = 1.93, *p* < 0.001); conversely, when presented with information emphasizing user-referent cues, participants rated the user dimension higher (M_item_ = 4.08, SD = 1.76; M_user_ = 6.12, SD = 0.76, *p* < 0.001). These results indicate that the manipulation of AI shopping assistants and algorithmic recommendation cues in Study 1 was successful.

We used SPSS 26.0 and AMOS 21.0 for the tests. At the 95% confidence interval level, the Cronbach’s α value for purchase intention was 0.841, CR was 0.842, AVE was 0.572, and all standardized factor loadings were greater than 0.5, indicating that the scale had good reliability and convergent validity.

**Figure 2:**
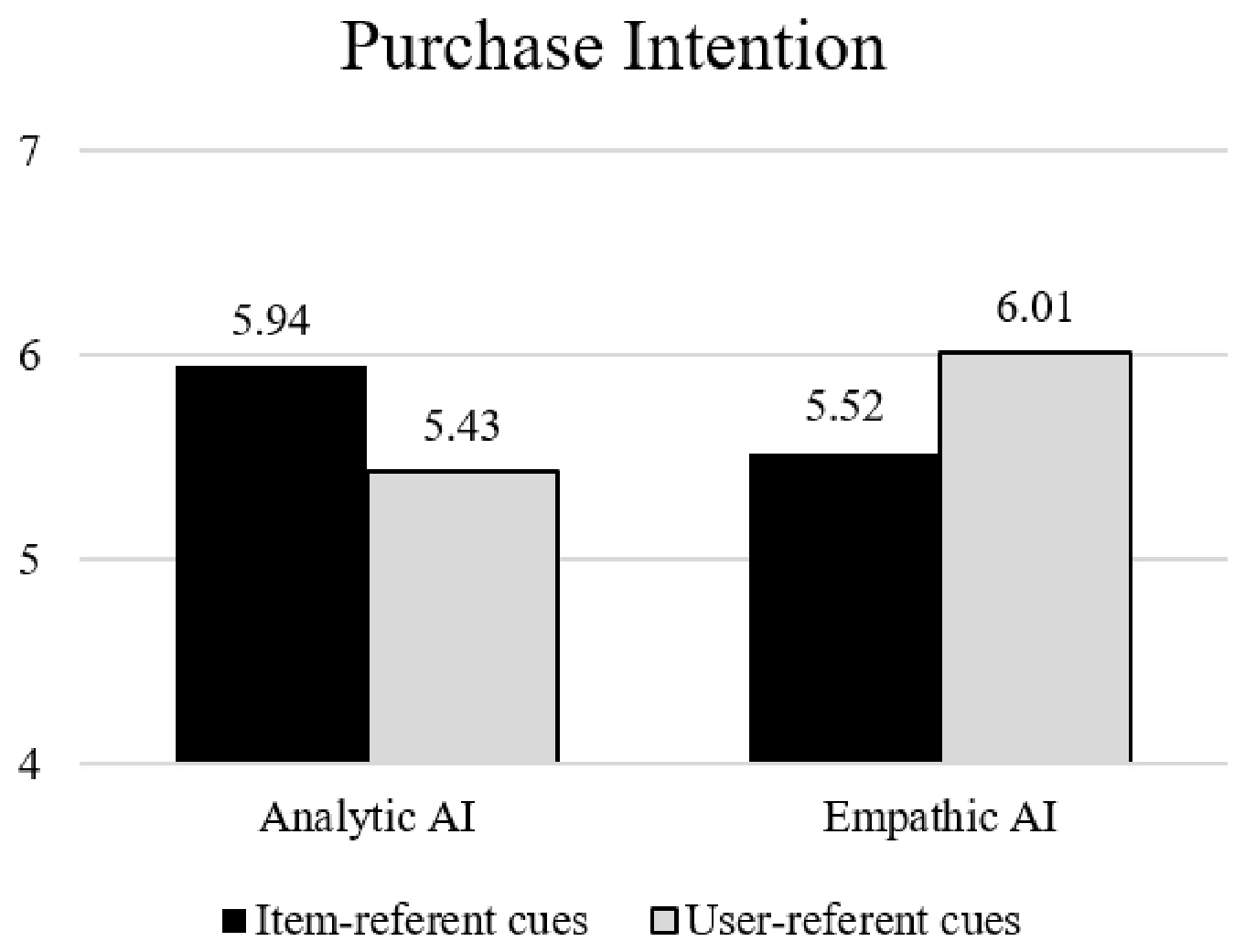
The interaction effect of AI shopping assistants and algorithmic recommendation cues (Study 1).

Study 1 demonstrated a significant interaction impact between AI shopping assistants and algorithmic recommendation cues using a two-way ANOVA with customers’ intentions to purchase organic food as the dependent variable (F(1, 259) = 50.57, *p* < 0.001, $\eta_{p}^{2}=0.163$). Furthermore, an independent-samples *t*-test revealed that information from analytic AI emphasizing item-referent cues (vs. user-referent cues) was more effective at stimulating purchase intention (M_analytic-item_ = 5.94, SD = 0.58; M_analytic-user_ = 5.43, SD = 0.61; t(130) = 4.92, *p* < 0.001, Cohen’s d = 0.85; see Figure 2). In contrast, information provided by the empathic AI that emphasized user-referent cues (vs. item-referent cues) was more effective at promoting organic food purchase intention (M_empathic-item_ = 5.52, SD = 0.49, M_empathic-user_ = 6.01, SD = 0.57, t(129) = −5.16, *p* < 0.001, Cohen’s d = 0.92; see Figure 2). H1 was confirmed.

We further examined the robustness of the interaction effect by including age as a continuous variable and gender, educational level, and monthly income as categorical control variables. After simultaneously adjusting for these demographic variables, the interaction between AI shopping assistants and algorithmic recommendation cues remained significant (F(1, 249) = 49.39, *p* < 0.001, $\eta_{p}^{2}=0.166$).

## 4. Study 2

### 4.1. Participants and Design

Study 2 is designed to examine the interaction effect of AI shopping assistants and algorithmic recommendation cues, as well as the mediating effect of perceived information validity and green trust, and their serial mediating role, specifically testing Hypotheses 1, 2, 3, and 4. We adopt a 2 × 2 factorial between-subject experimental design. We conducted a priori power analysis to determine the required minimum sample size. Leveraging G*Power 3.1 to calculate the sample size, with an F-test, an effect size of 0.25, α error level is 0.05, and a statistical power of 0.8, the total sample size was 128 [89].

Based on the pilot test in Study 1, in Study 2, we used organic potatoes as the formal experimental stimulus. By manipulating different AI shopping assistants and algorithmic recommendation cues, we examined the differences in consumers’ organic food purchase intentions after being exposed to different experimental materials. In the design of AI shopping assistants, similar to Study 1, we used the same images under different experimental conditions and distinguished the characteristics of different AI assistants through textual descriptions to eliminate the interference and noise caused by preferences and aversions to different external images (see the Appendix A). For the design of algorithmic recommendation cues, Like in Study 1, we employed two kinds of cues: item-referent and user-referent. Item-referent cues emphasize foods similar to products that consumers have previously shown interest in or browsed, while user-referent cues emphasize the purchase choices and preferences of similar consumers (see the Appendix A). Thus, four different experimental conditions were designed.

Similarly, we also set up corresponding manipulation check items in Study 2. After reading the stimulus material and completing the questions about purchase intentions, participants need to further answer the manipulation check questions. All items are designed on a 7-point scale (1 = strongly disapprove, 7 = strongly approve). The items regarding AI shopping assistants and algorithmic recommendation cues are the same as in Study 1.

Study 2 recruited participants through Credamo (a professional research platform). The study participation invitations were distributed to registered members, who voluntarily decided whether to participate. The sample for Study 2 should contain consumers who may encounter AI recommendations when purchasing food on shopping websites. Therefore, consumers of different genders, ages, education levels, and income groups were recruited without imposing population quotas that restrict the breadth of the sample. According to the pilot test, using organic potatoes, a food with high familiarity, makes the experimental context relevant to consumers. As shown in Table 4, the sample includes participants from various demographic categories.

Study 2, after obtaining informed consent, randomly assigned participants through the survey system with equal probability to one of the four experimental conditions. Multiple submissions and forwarding of the questionnaire are prohibited. A total of 280 questionnaires were collected. After excluding 2 patterned responses (such as all identical options and “Z”-shaped options) and 3 responses that met the criterion of a too-short completion time (60 s), 275 valid data points were retained (N_female_ = 67.27%, M_age_ = 30.17, SD = 9.37, N_analytic-item group_ = 70, N_analytic-user group_ = 69, N_empathic-item group_ = 67, N_empathic-user group_ = 69).

### 4.2. Procedure and Measures

At the beginning of the formal experiment, we set up a hypothetical scenario and informed the participants to imagine themselves in that scenario. We presented the following text: “Please imagine that you intend to purchase some potatoes and are browsing an online shopping platform. This platform will prioritize matching you with the nearest delivery station and deliver the food you ordered to your home within half an hour. The platform has set up AI shopping assistants that can recommend products to consumers based on relevant information in the shopping system. At this point, the AI shopping assistants recommended organic potatoes to you, and you saw the following information.” Then, participants were assigned to one of four experimental conditions at random.

In the formal experiment, we included additional information about organic potatoes to enhance the product’s authenticity. For example, we mentioned the following: “Certified as an organic product; certification details are available upon request,” and “Grown in an organically certified planting base; field management is conducted using crop rotation, manual weeding, and physical pest control methods.” Following the presentation of the information, participants were asked to score on a 7-point scale how likely they were to buy these organic potatoes (1 = strongly disapprove, 7 = strongly approve): (1) “It is very likely that I will purchase organic potatoes listed above;” (2) “I think the organic potatoes listed above is worth buying;” (3) “I would recommend the organic potatoes listed above to people I know;” (4) “Generally, I am willing to purchase the organic potatoes listed above.” (adapted from Li et al. [87]). In addition, participants were asked to rate their perceived information validity and green trust.

The items for perceived information validity include four statements: (1) “The recommendation information provided by this AI shopping assistant is generally truthful;” (2) “The recommendation information provided by this AI shopping assistant leaves me feeling accurately informed about this organic food product;” (3) “The recommendation information provided by this AI shopping assistant is believable;” (4) “The recommendation information provided by this AI shopping assistant is authentic rather than fabricated.” (adapted from Appelman and Sundar [90] and Haupt et al. [91]). The items regarding green trust include four: (1) “I believe the green claims and guarantees about this product are generally reliable;” (2) “I trust that this product can deliver on its green promises;” (3) “I think the product’s green reputation is trustworthy;” (4) “Overall, I am satisfied with the green considerations of this product.” (adapted from Huang et al. [28] and Chen [92]). Participants then answered the manipulation check items. Finally, they reported on demographic variables, including educational level, age, and gender.

Study 2 repeated the reliability, manipulation-check (paired-sample *t*-test), two-way ANOVA, and independent-samples *t*-test used in Study 1. H2 and H3 were tested separately using the PROCESS plugin (Version 4.0) for SPSS 26.0, choosing Model 8, with AI shopping assistant as X, algorithmic recommendation cue as W, perceived information validity or green trust as M, and purchase intention as Y. H4 was tested using Model 85, with perceived information validity as M1 and green trust as M2. The AI shopping assistant was coded as 1 = analytic AI and 2 = empathic AI; the recommendation cue was coded as 1 = item-referent and 2 = user-referent. All conditional process analyses were estimated using ordinary least squares regression and 5000 bootstrap resamples. We report coefficients, standard errors, t values, *p* values, and 95% confidence intervals. A conditional indirect effect was considered significant when its bootstrap confidence interval excluded 0.

### 4.3. Results

We first examined whether the experimental manipulation was successful. The paired-sample *t*-test findings demonstrated that when consumers received information from an analytic AI, they were more likely to perceive AI shopping assistants as the analytic type rather than the empathic type (M_analytic_ = 5.95, SD = 0.68; M_empathic_ = 4.17, SD = 1.43, *p* < 0.001); conversely, when receiving information from empathic AI, participants were more likely to perceive AI shopping assistants as the empathic type (M_analytic_ = 4.75, SD = 1.35; M_empathic_ = 5.71, SD = 0.88, *p* < 0.001). Regarding judgments of algorithmic recommendation cues, when participants received information emphasizing item-referent cues, they rated the item dimension higher (M_item_ = 5.80, SD = 0.87; M_user_ = 4.47, SD = 1.81, *p* < 0.001); conversely, when presented with information emphasizing user-referent cues, participants rated the user dimension higher (M_item_ = 4.33, SD = 1.71; M_user_ = 5.91, SD = 0.90, *p* < 0.001). These results indicate that the manipulation of AI shopping assistants and algorithmic recommendation cues in Study 2 was successful.

We used SPSS 26.0 and AMOS 21.0 for the tests. At the 95% confidence interval level, the Cronbach’s α values for purchase intention, perceived information validity, and green trust were 0.864, 0.828, and 0.827, respectively; the CR values were 0.866, 0.831, and 0.827, respectively; and the AVE values were 0.619, 0.553, and 0.545, respectively. All standardized factor loadings were greater than 0.5, meeting the criteria for convergent validity. Furthermore, we conducted a test of discriminant validity and found that the square roots of the AVE for each construct were greater than the correlations between the constructs, indicating that the constructs have good discriminant validity (Table 5). In addition, we also evaluated the goodness of fit of the model and found that the model fit well (χ^2^/df = 1.282, CFI = 0.992, TLI = 0.990, RMSEA = 0.032, SRMR = 0.027).

**Figure 3:**
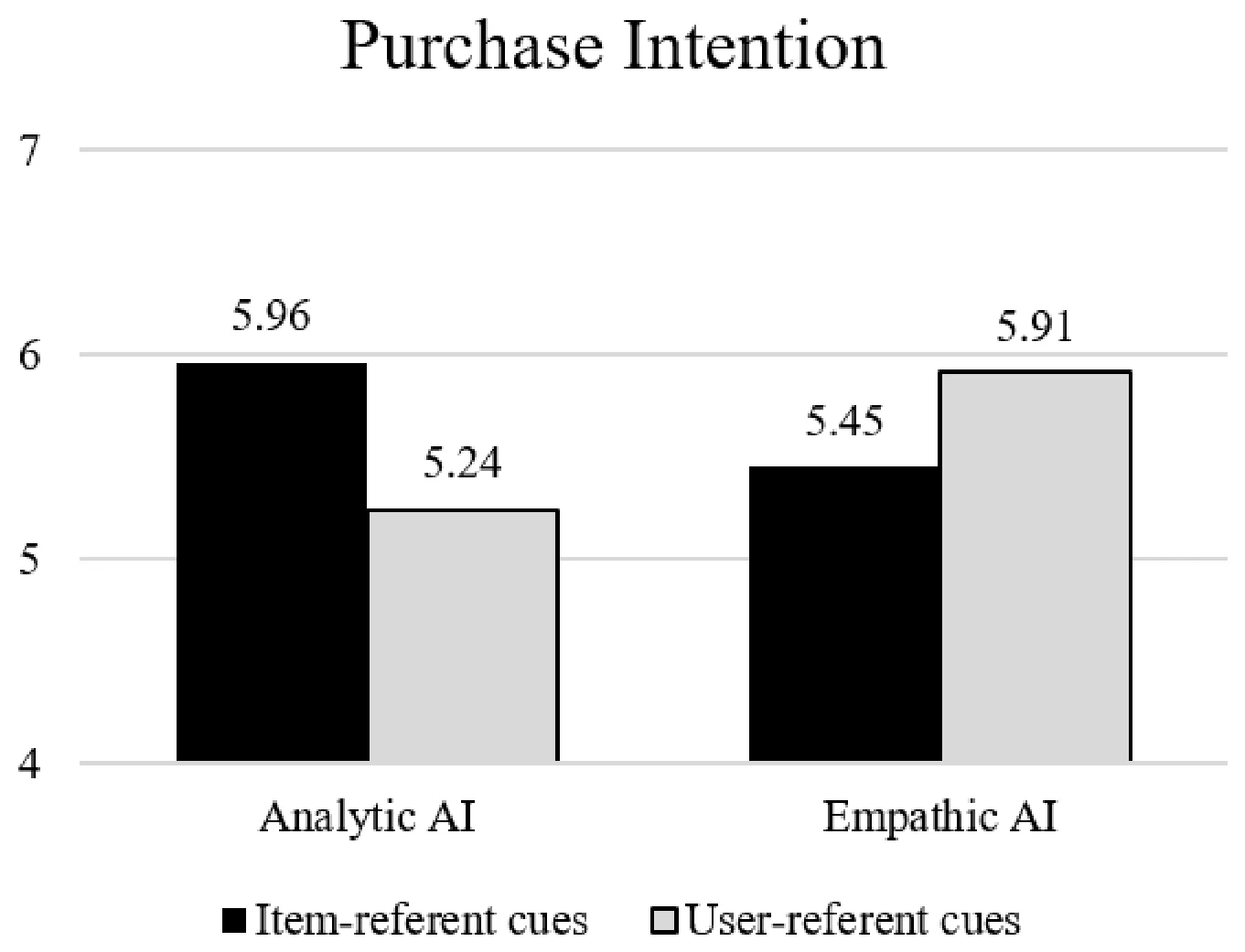
The interaction effect of AI shopping assistants and algorithmic recommendation cues (Study 2).

Study 2 demonstrated a significant interaction impact between AI shopping assistants and algorithmic recommendation cues using a two-way ANOVA with customers’ intentions to purchase organic food as the dependent variable (F(1, 271) = 66.87, *p* < 0.001, $\eta_{p}^{2}=0.198$). Furthermore, an independent-samples *t*-test revealed that information from analytic AI emphasizing item-referent cues (vs. user-referent cues) was more effective at stimulating purchase intention (M_analytic-item_ = 5.96, SD = 0.57; M_analytic-user_ = 5.24, SD = 0.73; t(137) = 6.43, *p* < 0.001, Cohen’s d = 1.10; see Figure 3). In contrast, information provided by the empathic AI that emphasizes user-referent cues (vs. item-referent cues) was more effective at promoting organic food purchase intention (M_empathic-item_ = 5.45, SD = 0.55, M_empathic-user_ = 5.91, SD = 0.52, t(134) = −5.08, *p* < 0.001, Cohen’s d = 0.86; see Figure 3). H1 was again validated.

The robustness analysis was conducted for Study 2. Age was included as a continuous variable, whereas gender, educational level, and monthly income were included as categorical control variables. The interaction between AI shopping assistants and algorithmic recommendation cues remained significant after simultaneously adjusting for these demographic variables (F(1, 261) = 64.18, *p* < 0.001, $\eta_{p}^{2}=0.197$).

We then examined the mediating role of perceived information validity. Using the PROCESS plugin for SPSS (Version 4.0) to conduct a bootstrap analysis, at the 95% confidence interval, we chose Model 8 and used 5000 bootstrap resamples. The findings demonstrated that the interaction term between AI shopping assistants and algorithmic recommendation cues had a positive effect on purchase intention (β = 0.68, t = 6.62, SE = 0.10, CI = [0.48, 0.88], *p* < 0.001, excluding 0) and perceived information validity (β = 0.72, t = 4.75, SE = 0.15, CI = [0.42, 1.01], *p* < 0.001, excluding 0). The effect of perceived information validity on purchasing intention was also significant (β = 0.70, t = 17.61, SE = 0.04, CI = [0.62, 0.78], *p* < 0.001, excluding 0). Results were presented in Table 6, thereby demonstrating the mediating effect of perceived information validity. Further analysis of the mediating role of perceived information validity in response to different algorithmic recommendation cues (Table 7) revealed that in the item-referent cues group, perceived information validity had a significant mediating effect on the influence of AI shopping assistants on purchase intention (CI = [−0.387, −0.077], excluding 0); in the user-referent cues group, the mediating effect of perceived information validity remained significant (CI = [0.129, 0.460], excluding 0), and H2 was confirmed.

In addition, Study 2 examined the mediating role of green trust. A bootstrap analysis was conducted using the PROCESS plugin for SPSS (Version 4.0), choosing Model 8 and 5000 bootstrap resamples, establishing a 95% confidence interval. The findings demonstrated that the interaction term between AI shopping assistants and algorithmic recommendation cues had a positive effect on purchase intention (β = 0.61, t = 6.02, SE = 0.10, CI = [0.41, 0.81], *p* < 0.001, excluding 0) and green trust (β = 0.78, t = 5.30, SE = 0.15, CI = [0.49, 1.07], *p* < 0.001, excluding 0). The effect of green trust on purchase intention was also significant (β = 0.72, t = 18.17, SE = 0.04, CI = [0.64, 0.80], *p* < 0.001, excluding 0). Results were presented in Table 8, thereby confirming the mediating effect of green trust. Further analysis of green trust’s mediating role in response to different algorithmic recommendation cues (Table 9) revealed that in the item-referent cues group, green trust had a significant mediating effect on the influence of AI shopping assistants on purchase intention (CI = [−0.420, −0.107], excluding 0); in the user-referent cues group, the mediation of green trust remained significant (CI = [0.147, 0.508], excluding 0), and H3 was confirmed.

**Table 8:** Test results for the mediating effect of green trust (Model 8).

Dependent Variable	Independent Variable	Coeff.	SE	t	LLCI	ULCI
Green trust	AI shopping assistants	−1.14	0.23	−4.87 ***	−1.60	−0.68
	Algorithmic recommendation cues	−1.24	0.23	−5.34 ***	−1.70	−0.79
	Int_1	0.78	0.15	5.30 ***	0.49	1.07
Purchase intention	AI shopping assistants	−0.86	0.16	−5.40 ***	−1.18	−0.55
	Algorithmic recommendation cues	−0.99	0.16	−6.19 ***	−1.31	−0.68
	Int_1	0.61	0.10	6.02 ***	0.41	0.81
	Green trust	0.72	0.04	18.19 ***	0.65	0.80

Note: * *p* < 0.05, ** *p* < 0.01, *** *p* < 0.001; Int_1 refers to the interaction between AI shopping assistants and algorithmic recommendation cues.

**Table 9:** Test results for the mediating role of green trust under different levels of algorithmic recommendation cues.

Algorithmic Recommendation Cues	Effect	SE	LLCI	ULCI
Item-referent cues	−0.258	0.079	−0.420	−0.107
User-referent cues	0.310	0.093	0.147	0.508

**Figure 4:**
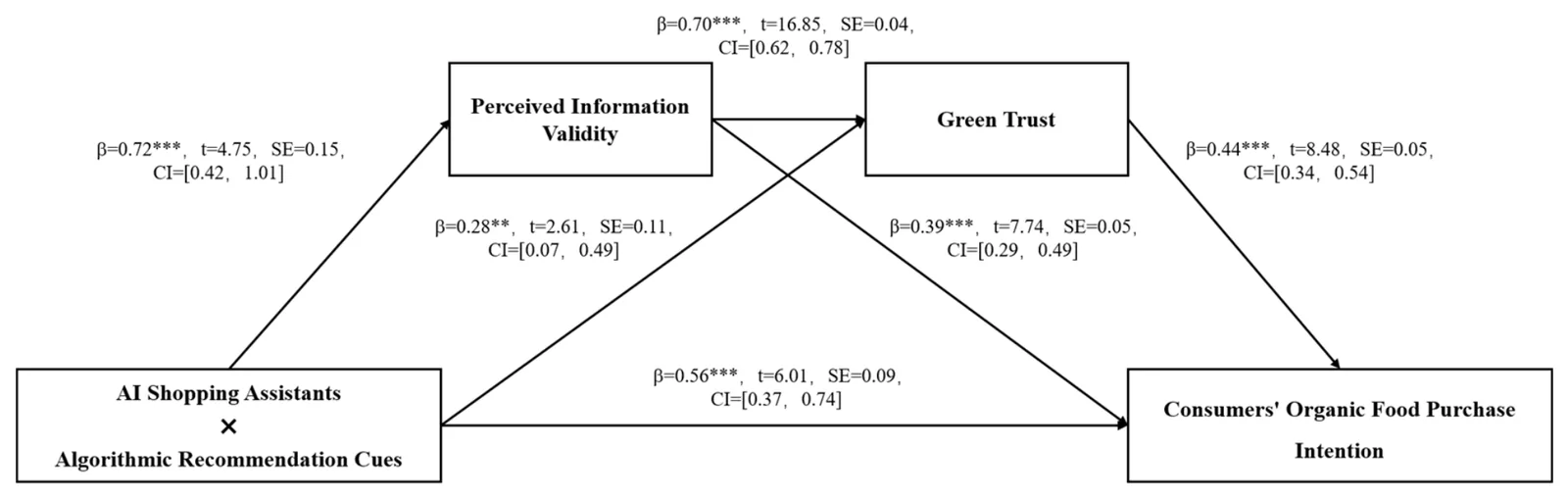
Serial mediating role of perceived information validity and green trust. Note: A conditional serial mediation model estimated using Model 85 in the PROCESS plugin for SPSS (Version 4.0). Values are ordinary least squares regression coefficients. Corre-sponding standard errors, t values, *p* values, and 95% confidence intervals are reported in the results, and the conditional serial indirect effects based on 5000 bootstrap resamples are report-ed in Table 10. * *p* < 0.05, ** *p* < 0.01, *** *p* < 0.001.

**Figure 5:**
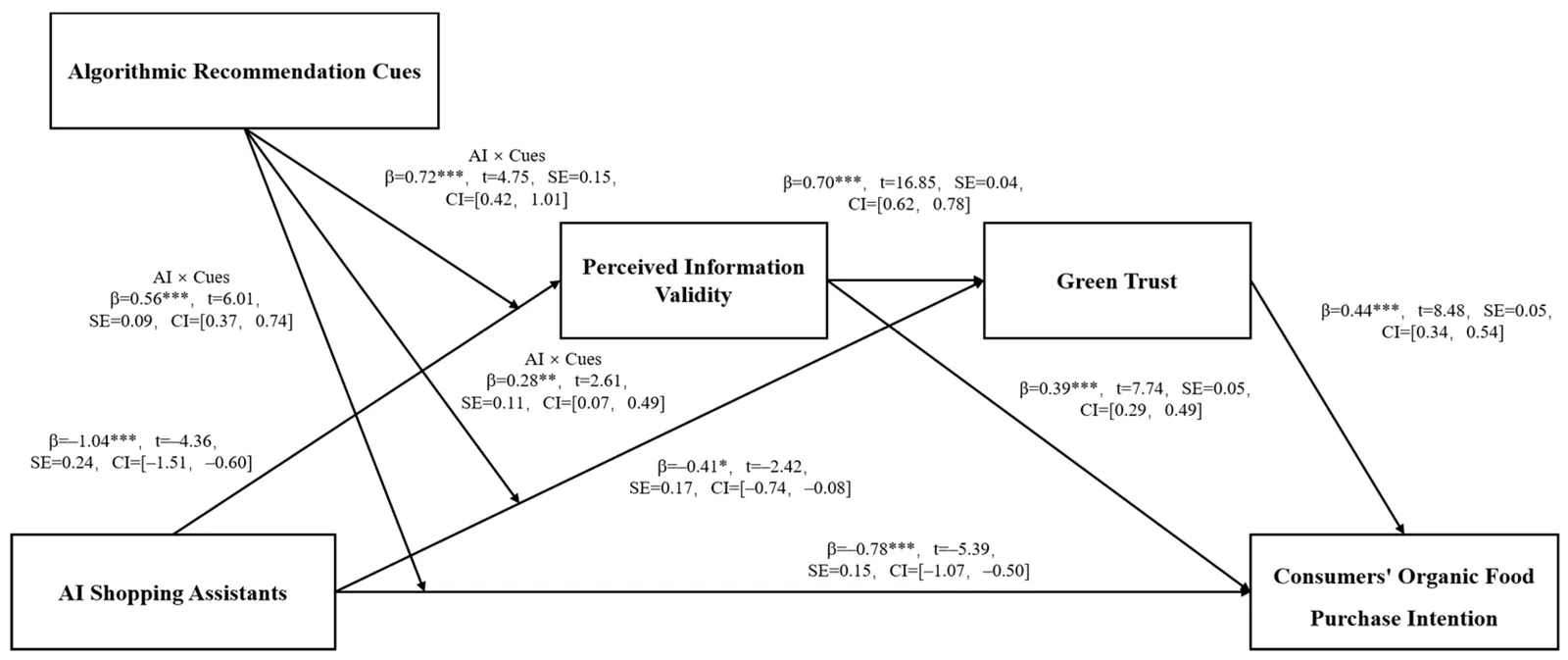
Serial mediating role of perceived information validity and green trust (Model 85). Note: * *p* < 0.05, ** *p* < 0.01, *** *p* < 0.001.

Further, Study 2 examined the serial mediating effect of perceived information validity and green trust. A bootstrap analysis was conducted using the PROCESS plugin for SPSS (Version 4.0), choosing Model 85 and 5000 bootstrap resamples, establishing a 95% confidence interval. The findings demonstrated that the interaction term between AI shopping assistants and algorithmic recommendation cues had a positive effect on purchase intention (β = 0.56, t = 6.01, SE = 0.09, CI = [0.37, 0.74], *p* < 0.001, excluding 0), perceived information validity (β = 0.72, t = 4.75, SE = 0.15, CI = [0.42, 1.01], *p* < 0.001, excluding 0), and green trust (β = 0.28, t = 2.61, SE = 0.11, CI = [0.07, 0.49], *p* < 0.01, excluding 0), and perceived information validity had an effect on purchase intention (β = 0.39, t = 7.74, SE = 0.05, CI = [0.29, 0.49], *p* < 0.001, excluding 0) and green trust (β = 0.70, t = 16.85, SE = 0.04, CI = [0.62, 0.78], *p* < 0.001, excluding 0), and green trust had a similarly significant positive effect on purchase intention (β = 0.44, t = 8.48, SE = 0.05, CI = [0.34, 0.54], *p* < 0.001, excluding 0), thereby demonstrating the serial mediating effect of perceived information validity and green trust (see Figure 4). We also presented a more precise path diagram for Model 85 (Figure 5). Further analysis of the serial mediating role across different algorithmic recommendation cues (Table 10) revealed that in the item-referent cues group, perceived information validity and green trust exhibited a significant serial mediating effect on the influence of AI shopping assistants on purchasing intentions (CI = [−0.190, −0.028], excluding 0); in the user-referent cues group, the significance of the serial mediating effect persisted (CI = [0.044, 0.238], excluding 0), and H4 was confirmed.

**Table 10:** Test results for the serial mediating role under different levels of algorithmic recommendation cues.

Algorithmic Recommendation Cues	Effect	SE	LLCI	ULCI
Item-referent cues	−0.099	0.042	−0.190	−0.028
User-referent cues	0.120	0.050	0.044	0.238

The primary PROCESS models did not include demographic variables. In supplementary robustness analyses, age was entered as a continuous variable, whereas gender, educational level, and monthly income were coded and entered as categorical variables. All conditional process models were estimated using ordinary least squares regression and 5000 bootstrap resamples. The primary coefficient-level tests used conventional OLS standard errors, and indirect effect inference was based on 95% bootstrap confidence intervals. Heteroscedasticity-consistent HC3 standard errors were additionally used as a sensitivity check. A conditional indirect effect was considered statistically significant when its bootstrap confidence interval excluded 0.

The robustness analyses produced the same inferential conclusions. After age, gender, educational level, and monthly income were included, the conditional indirect effect through perceived information validity remained significant under both item-referent cues (β = −0.212, CI = [−0.362, −0.069], excluding 0) and user-referent cues (β = 0.275, CI = [0.127, 0.455], excluding 0). The corresponding indirect effects through green trust were also significant under item-referent cues (β = −0.244, CI = [−0.403, −0.098], excluding 0) and user-referent cues (β = 0.302, CI = [0.145, 0.491], excluding 0). Finally, the conditional serial mediating effects remained significant under item-referent cues (β = −0.089, CI = [−0.173, −0.025], excluding 0) and user-referent cues (β = 0.116, CI = [0.045, 0.219], excluding 0). In addition, the use of HC3 standard errors did not change the significance of any focal path in Model 8 or Model 85 (The effect of the interaction term on green trust is β = 0.281, SEHC3 = 0.105, *p* < 0.01; all other core paths have *p* < 0.001).

The obtained results are theoretically supported by the Stereotype Content Model and Cue Utilization Theory. The Stereotype Content Model explains why consumers interpret analytic and empathic AI through competence and warmth, respectively. Cue Utilization Theory further suggests that these cues are more diagnostic when aligned with the recommendation content: analytic AI enhances the diagnosticity of product-related evidence, while empathic AI strengthens the relevance of user-related social evidence. The mediation results are also consistent with Signaling Theory, as AI characteristics and recommendation rationales serve as observable signals for otherwise unverified organic and environmental attributes. Consumers assess these signals for validity, which shapes green trust and purchase intention, consistent with the observed serial mediation pathway.

## 5. Discussion

This work finds that AI shopping assistants and algorithmic recommendation cues do not function independently; rather, they influence consumers’ organic food purchase intention through matched interactions. Analytic AI is more effective when combined with item-referent cues, while empathic AI performs better when combined with user-referent cues. This result is consistent with the research conclusion that emphasizes the contextual advantages of different AI types [93]. They found that in the context of online food shopping, ability-oriented AI can enhance perceived value more effectively when consumers engage in systematic information processing, while warmth-oriented AI is more effective under heuristic processing conditions, with perceived match playing a mediating role. Similar to the conclusions of our work, Zhu et al. [53] found that in service contexts primarily focused on functionality and problem-solving, consumers prefer expert AI that emphasizes professional competence, whereas in contexts that emphasize experience and relational value, consumers prefer partner AI that embodies warmth and companionship. Moreover, the comparison between empathic and mechanical chatbots by Juquelier et al. [94] also indicates that empathic AI can improve consumer experience by enhancing social presence, which is consistent with the findings of this work that empathic AI can enhance persuasion effectiveness by strengthening social connection. These studies collectively indicate that neither analytic AI nor empathic AI possesses an absolute advantage outside of context; their effectiveness depends on the consistency between the type of AI and the current decision task, information processing method, and evidence structure. Research on recommendation systems also indicates that item-based and user-based recommendations are not functionally equivalent methods, and consumer preferences for the two can vary depending on the source of information and the basis of judgment [19]. This aligns with the conclusion of this work regarding the combination of different AI assistants with different algorithmic recommendation cues to enhance persuasive effects. These findings extend the work of Nozawa et al. [26], who show that consumers evaluate AI-involved restaurants less favorably than human ones, especially in luxury settings, due to lower expected food, service, and ambience quality. Their results suggest that responses to AI in food contexts depend on the experiential and hedonic nature of the setting. The present study complements this by showing that consumer responses also vary across AI types. We distinguish between analytic and empathic AI and show that their effectiveness depends on algorithmic recommendation cues. Moreover, Nozawa et al. [26] focused on restaurant quality expectations and visit intentions, we identify perceived information validity and green trust as sequential mechanisms driving organic food purchase intention. Overall, these findings indicate that AI effectiveness in food decisions depends on the fit between AI capabilities and contextual evaluative demands.

Nevertheless, this study does not fully support the view that user-based framing has a universal advantage. Gai and Klesse [18] found that presenting the same recommendation as user-based framing generally achieves a higher click-through rate compared to item-based framing. Zhang et al. [61] also found that normative recommendation frames based on others’ choices are generally more effective than those based on product comparisons. In contrast, this study shows that when the information source is analytic AI, item-referent cues are more effective in enhancing purchase intention. This difference may stem from the fact that existing studies typically fix the information source, mainly comparing the recommendation cue framework itself, and thus do not examine the capabilities and emotional characteristics presented by different information sources. Moreover, the aforementioned studies mostly focus on general products, with their outcome variables often being click intentions. In contrast, this research focuses on organic food, where the purchase involves price premiums and trust attributes such as authenticity, safety, and environmental properties that are difficult to directly verify, requiring consumers to make more in-depth judgments about the recommendation evidence. At this point, the social recognition provided by user-referent cues is not necessarily superior; item-referent cues, when combined with the professional capabilities and logical analysis of analytic AI, are more diagnostic and persuasive.

In terms of the mechanism, this work finds that perceived information validity and green trust not only mediate the interaction effects of AI shopping assistants and algorithmic recommendation cues, respectively, but also form a serial mediating effect. First, the mediating role of perceived information validity is highly consistent with the findings of Hundt and Seta [95] on information judgment. They point out that consumers’ judgment of information validity depends on the credibility of the information source, indicating that the content of the information is not evaluated in isolation. The characteristics of the source and the structure of the evidence are integrated to jointly determine whether the information can be trusted. Anderl et al. [96] on AI communication also indicate that the way information is presented and the accuracy of the information itself will jointly affect consumers’ credibility judgments. However, Pham and Nguyen [97] found that the information quality of voice assistants can promote purchase intention through perceived validity, but the indirect effect of information credibility on purchase intention via perceived validity is not significant, which is not entirely consistent with the findings of this study that perceived information validity has a stable mediating effect. The reason may lie in the fact that voice shopping typically has immediacy and transaction orientation, and factors such as price, convenience, and product functionality may weaken the independent effect of information credibility. In contrast, the organic attributes, production process, and environmental commitments of organic food are difficult to directly verify before purchase, leading consumers to rely more on the accuracy and reasonableness of recommendation information to reduce uncertainty. Secondly, the mediating role of green trust is supported by the research of Roh et al. [71], which found that consumers’ perceived green value and product knowledge can enhance green trust, further promoting organic food purchase intention. Finally, the chain mediation results of this study align with the mediation sequence revealed by Fu et al. [98], indicating that consumers are more likely to convert their judgment of the information into trust in the product’s organic attributes and green commitments only after confirming the validity of the recommended information, thereby increasing the purchase intention of green agricultural products. Thus, this study further reveals the progressive mechanism from information validity perception to green trust and finally to purchase intention in the context of AI recommendations.

## 6. Conclusions

### 6.1. Research Findings

This work examines the interaction effect of AI shopping assistants (analytic AI vs. empathic AI) and algorithmic recommendation cues (item-referent cues vs. user-referent cues) on consumers’ organic food purchasing intentions, and tests the mediating effects of perceived information validity and green trust, as well as their serial mediating role. The research finds the following: (1) AI shopping assistants and algorithmic recommendation cues interact to affect intentions to buy organic food; analytic AI assistants that convey item-referent cues (vs. user-referent cues) are more effective at enhancing purchase intention, while empathic AI assistants perform better when combined with user-referent cues (vs. item-referent cues). (2) Perceived information validity mediates the interaction effect between AI shopping assistants and algorithmic recommendation cues. (3) Green trust mediates the interaction effect between AI shopping assistants and algorithmic recommendation cues. (4) Perceived information validity and green trust play a significant serial mediating role; when consumers perceive information validity, it further enhances green trust, which influences purchase intention.

### 6.2. Theoretical Implications

First, this work expands the research perspective on AI shopping assistants in the field of organic food consumption, advancing the research question from a static comparison of which type of AI is more effective to a dynamic comparison of which type of AI is more effective under which recommendation method. Existing research primarily examines the types of AI intelligence or social roles and their impact on consumer responses, which tends to create a comparative research logic between the effectiveness of analytic AI and empathic AI [99]. Although existing research has begun to find that the effects of capability-oriented and warmth-oriented AI vary with the customer decision-making stage [53,93], there is still a lack of systematic explanation on how AI capability characteristics and recommendation rationale evidence cues jointly shape consumer judgment. This work integrates AI shopping assistants with algorithmic recommendation cues into a unified framework, indicating that the two types of AI do not possess absolute advantages outside of context. Analytic AI works better when paired with item-referent cues, whereas empathic AI works better when paired with user-referent cues. Therefore, this work expands the unit of analysis in AI marketing research from a single AI feature to the configuration relationship between AI capability features and algorithmic recommendation cues, and reveals the contextual boundaries of how different types of AI shopping assistants influence organic food purchase intention.

Second, this work expands the applicability of Cue Utilization Theory in the field of sustainable food consumption by revealing the interaction effect between AI source characteristics and algorithmic recommendation cues, and establishes a theoretical link between it and the Stereotype Content Model. Traditional cue utilization research primarily compares the relative impact of intrinsic and extrinsic cues within products, emphasizing that consumers judge product quality based on the diagnosticity of these cues [49]. Some studies further indicate that digital marketing cues are not independent of each other, and their effects are influenced by other cues and the usage context [50,100]. This work further distinguishes between AI assistants as source cues and algorithmic recommendation cues as evidence cues, using the Stereotype Content Model to explain how consumers form competence and warmth perceptions from AI’s performance and characteristics [47]. Cue Utilization Theory further explains how these source cues interact with product-matching or user-matching cues to enhance purchase intention. The capabilities and objectivity of analytic AI enhance the diagnostic value of item-referent cues, while the warmth, understanding, and empathy of empathic AI strengthen the social connection and individual relevance conveyed by user-referent cues. Thus, this work extends Cue Utilization Theory from the comparison of cues at the same information level to the cross-level matching of source cues and evidence cues, and explains that cue diagnosticity is not a fixed attribute, but depends on the semantic consistency between different cues and the fluency with which consumers process these cues. This deepens the understanding of the shift in AI persuasion from the independent role of multiple cues to their matching role.

Third, this work refines the explanation of Signaling Theory for AI-driven sustainable food consumption through the mediating role of perceived information validity and green trust, particularly the serial mediating effect between the two. Signaling Theory typically explains how information senders use observable signals to reduce information asymmetry, allowing receivers to infer product quality that is difficult to directly observe [77]; research on sustainable consumption also primarily views green attributes or environmental information transparency as signals that form consumer trust [80,98]. This work considers AI shopping assistants and algorithmic recommendation cues as composite signals and reveals the process of receiving and processing these signals. The significant indirect effects are consistent with a theoretically ordered association from information-validity judgment to green trust and purchase intention. The perceived information validity positively influences green trust, and green trust positively promotes organic food purchase intention. Therefore, this work advances Signaling Theory from a simplified relationship where signals directly trigger behavior to a sequential model that includes cognitive validation and trust conversion and endows AI shopping assistants with the multiple theoretical roles of signal transmitters and interpreters.

### 6.3. Practical Implications

This work also provides practical implications. First, the interaction effects indicate that marketers should not view analytic AI or empathic AI as universally superior choices but should configure them around AI capability characteristics and algorithmic recommendation cues. Related food marketing research also indicates that the alignment between the recommender and the information framework significantly affects consumer responses [87]. Under item-referent cues, companies can prioritize deploying analytic AI, using concise, professional, and evidence-based expressions, and presenting relevant information through attribute comparison cards. This includes details such as organic certification numbers, origin, cultivation methods, pesticide residue testing, nutritional components, and differences from similar products, making the reasons for recommending the product clear and visible. Under user-referent cues, empathic AI can be prioritized, incorporating information actively provided or authorized by the consumer, such as dietary preferences, health goals, allergy information, past purchase records, and preferences of similar users, to emphasize emotional and empathetic expressions explaining why the product is suitable for that consumer. If a company uses only one AI assistant, it can establish a switchable interaction module that dynamically adjusts tone, dialog scripts, and explanation templates based on recommendation criteria and continuously compare click-through rates, add-to-cart rates, conversion rates, and return rates through different combinations of AI types and recommendation cues. It is important to emphasize that this does not mean completely excluding non-matching combinations; rather, matching combinations should be set as the default priority strategy, while the persuasiveness of other combinations should be enhanced through subsequent information validity and trust design. The integration of AI and customer experience should also be based on contextualized configurations rather than a single technology deployment.

Second, the mediating role of perceived information validity means that marketers should shift their management focus from making AI’s expressions smoother to ensuring that the recommendations are verifiable. Research shows that providing explanations for AI recommendations can enhance consumers’ perception of explainability, which can further boost purchase intentions and behaviors [101]. Therefore, companies can establish a hierarchical evidence structure for recommendation information. For example, first provide a concise recommendation conclusion, then allow consumers to expand and view the evidence sources, data dates, measurement units, certification bodies, and applicable scope, and finally provide clickable test reports or supply chain traceability records. In item-referent cues, it should be ensured that there is a clear connection between product attributes and the recommendation conclusion; in user-referent cues, it should be explained which user preferences were invoked and how the basis for similarity was formed, and consumers should be allowed to modify, withdraw, or correct their personal information. Companies should also clearly distinguish between verified facts, algorithmic inferences, and algorithmic predictions, appropriately disclose the degree of confidence and information limitations, and avoid presenting speculation as established facts. In addition, the backend can further set up reminders for evidence expiration, detection of contradictory information, and manual review mechanisms and incorporate evidence view rates, information correction rates, and acceptance rates of recommended explanations into the algorithm recommendation metrics.

Last, the mediating role of green trust indicates that marketers need to further convert consumers’ one-time recognition of recommendations into sustained trust in organic attributes and green commitments. Companies should ensure that the green information in AI responses, product detail pages, packaging labels, and advertisements is consistent and provide external assurance for green claims through third-party organic certification, scannable traceability codes, test reports, supplier records, and regular audits. For expired certifications, changes in origin, or incorrect information, timely notifications and corrections should be made, and channels for manual consultation and dispute resolution should be provided to avoid a single instance of misinformation from damaging overall green credibility. Green trust is an important factor connecting green value judgment and organic food purchases, and information validity and green trust also impact customers’ willingness to make purchases. Based on the chain mediation results of this study, marketers can construct a reasonable sequential process. For example, they can first help consumers confirm the accuracy and validity of the recommended information, then present certifications, performance records, and third-party guaranties, and finally provide purchase access, quality commitments, or after-sales support. For consumers who have already viewed the evidence but have not yet formed trust, it is advisable to focus on supplementing certification and traceability information; for consumers who have formed trust but have not yet made a purchase, offering trial purchases, discounts, or return and exchange guaranties can be effective. In addition, companies should separately monitor perceived information validity, green trust, and purchase conversion rates to identify whether consumers are at the stage of unrecognized information or untrusted green commitments, thereby implementing more precise information interventions.

### 6.4. Limitations

This research still has the following limitations. First, it only distinguishes AI shopping assistants as analytic and empathic AI, without considering other or hybrid types. Future research could adopt a more fine grained classification of AI assistants. Second, both studies used online samples from China, with relatively young and well-educated participants and no cross-cultural comparison, which may limit generalizability. Future studies should use more diverse and cross-cultural samples. Third, the experiments relied on self-reported purchase intention rather than actual purchase behavior as the outcome variable, which may involve potential ceiling effects. Future research should use field designs with behavioral outcomes. Fourth, key mediators were measured at the same time point, lacking examination of the temporal causal order of perceived information validity, green trust, and purchase intention. Future studies should use longitudinal experimental designs to establish causal order. Fifth, only two organic products (milk and potatoes) were used, which may limit generalizability across product categories. Future research should test a wider range of products or manipulate product characteristics.

## Figures and Tables

**Table 1:** Demographic information of pilot test.

Type	Item	Value	Quota Proportion (%)
Gender	Male	35	31.82
	Female	75	68.18
Age	18–20	8	7.27
	21–30	51	46.36
	31–40	31	28.18
	41–50	10	9.09
	51–60	9	8.18
	Over 60	1	0.91
Educational level	Junior high school	1	0.91
	High school	6	5.45
	Associate degree	12	10.91
	Bachelor’s degree	78	70.91
	Master’s degree	12	10.91
	PhD	1	0.91

**Table 2:** Results of pilot test.

FAO Food Category	Representative Product	Familiarity (Mean)	Frequency of Consumption (Mean)	Purchase Likelihood (Mean)
Cereals	organic rice	5.76	5.19	5.96
Fats and oils	organic peanut oil	5.58	4.91	5.77
Sugar	organic white sugar	4.05	3.21	4.46
Fruit and vegetables	organic apples	5.68	4.90	5.81
Roots, tubers and pulses	organic potatoes	5.78	5.06	6.03
Meat	organic pork	4.86	4.12	5.37
Dairy and eggs	organic pure milk	6.08	5.52	6.15
Beverages and other	organic tea leaves	4.02	3.20	4.27

Note: Organic white sugar was included only to preserve coverage of the “sugar” category used in FAO food-supply classifications. Its inclusion as a candidate stimulus did not indicate a certain health recommendation or imply that greater sugar consumption is desirable. The results showed that organic white sugar received relatively low familiarity, consumption-frequency, and purchase-likelihood ratings and was therefore not selected for either formal study.

**Table 3:** Demographic information of Study 1.

Type	Item	Value	Quota Proportion (%)
Gender	Male	110	41.83
	Female	153	58.17
Age	18–20	20	7.60
	21–30	147	55.89
	31–40	65	24.71
	41–50	23	8.75
	51–60	4	1.52
	Over 60	4	1.52
Educational level	Junior high school	6	2.28
	High school	10	3.80
	Associate degree	24	9.13
	Bachelor’s degree	194	73.76
	Master’s degree	29	11.03

**Table 4:** Demographic information of Study 2.

Type	Item	Value	Quota Proportion (%)
Gender	Male	90	33.73
	Female	185	67.27
Age	18–20	21	7.64
	21–30	139	50.55
	31–40	85	30.91
	41–50	18	6.55
	51–60	9	3.27
	Over 60	3	1.09
Educational level	Junior high school	5	1.82
	High school	13	4.73
	Associate degree	29	10.55
	Bachelor’s degree	201	73.09
	Master’s degree	26	9.45
	PhD	1	0.36

**Table 5:** Discriminant validity analysis results.

Constructs	Perceived Information Validity	Green Trust	Purchase Intention
Perceived information validity	0.744		
Green trust	0.709	0.738	
Purchase intention	0.691	0.699	0.787

**Table 6:** Test results for the mediating effect of perceived information validity (Model 8).

Dependent Variable	Independent Variable	Coeff.	SE	t	LLCI	ULCI
Perceived information validity	AI shopping assistants	−1.04	0.24	−4.36 ***	−1.51	−0.57
	Algorithmic recommendation cues	−1.13	0.24	−4.76 ***	−1.60	−0.66
	Int_1	0.72	0.15	4.75 ***	0.42	1.01
Purchase intention	AI shopping assistants	−0.96	0.16	−5.96 ***	−1.28	−0.64
	Algorithmic recommendation cues	−1.10	0.16	−6.82 ***	−1.42	−0.78
	Int_1	0.68	0.10	6.62 ***	0.48	0.88
	Perceived information validity	0.70	0.04	17.61 ***	0.62	0.78

Note: * *p* < 0.05, ** *p* < 0.01, *** *p* < 0.001; Int_1 refers to the interaction between AI shopping assistants and algorithmic recommendation cues.

**Table 7:** Test results for the mediating role of perceived information validity under different levels of algorithmic recommendation cues.

Algorithmic Recommendation Cues	Effect	SE	LLCI	ULCI
Item-referent cues	−0.227	0.079	−0.387	−0.077
User-referent cues	0.274	0.086	0.129	0.460

## Data Availability

The raw data supporting the conclusions of this article will be made available by the authors on request.

## Appendix A

**Table A1:** Experimental Materials for Study 1.

Item	User

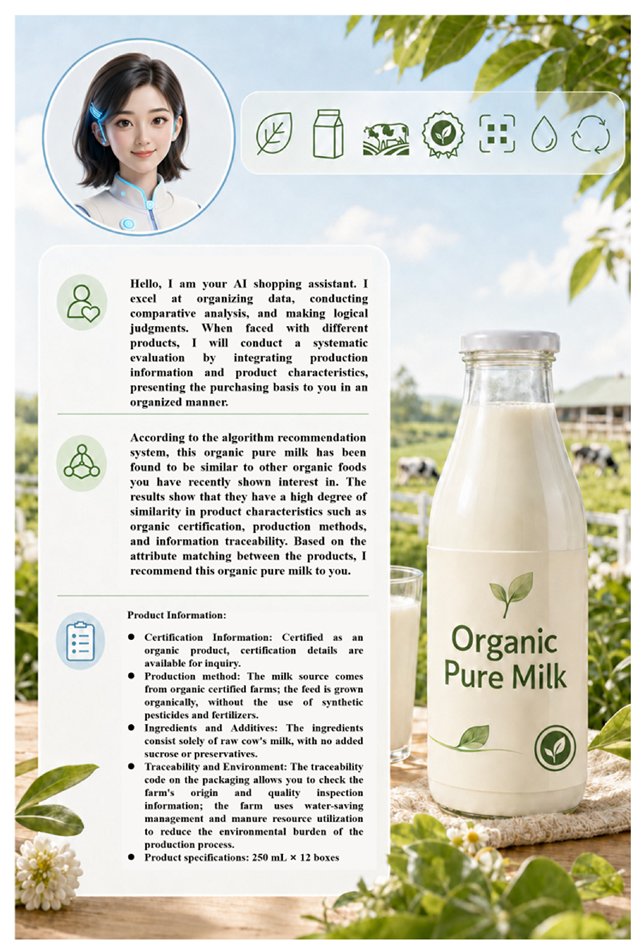
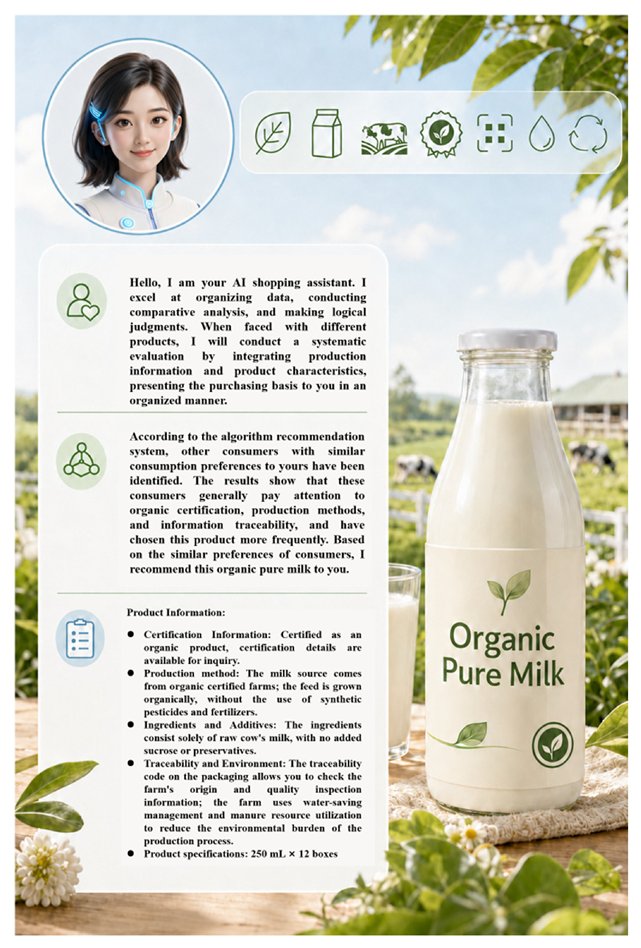

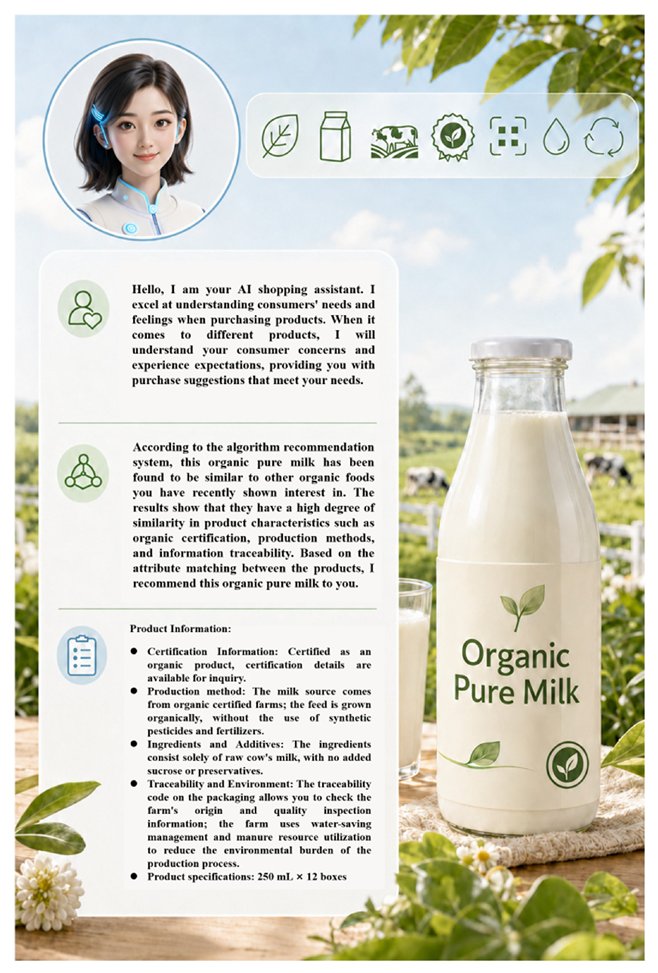
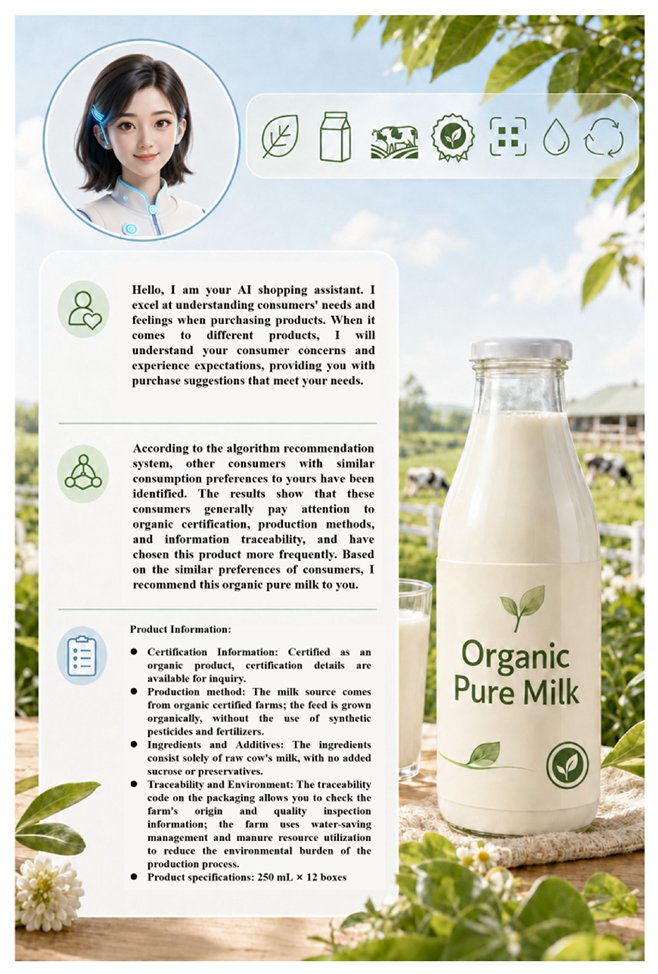
	
	

**Table A2:** Experimental Materials for Study 2.

Item	User
	
	
